# Advances in Adjoint Functions of Connection Number in Water Resources Complex Systems: A Systematic Review

**DOI:** 10.3390/e26040339

**Published:** 2024-04-16

**Authors:** Liangguang Zhou, Juliang Jin, Rongxing Zhou, Yi Cui, Chengguo Wu, Yuliang Zhou, Shibao Dai, Yuliang Zhang

**Affiliations:** 1 School of Civil Engineering, Hefei University of Technology, Hefei 230009, China; zhouliangguang@126.com (L.Z.); jinjl66@126.com (J.J.); zhangyuliang@hfut.edu.cn (Y.Z.); 2 School of Geographic Information and Tourism, Chuzhou University, Chuzhou 239000, China; 3 School of Environment and Energy Engineering, Anhui Jianzhu University, Hefei 230601, China; 4 College of Civil and Architecture, Chuzhou University, Chuzhou 239000, China

**Keywords:** water resource complex system, uncertainty analysis, connection number, adjoint function, set pair potential, partial connection number, connection entropy

## Abstract

The adjoint function of connection number has unique advantages in solving uncertainty problems of water resource complex systems, and has become an important frontier and research hotspot in the uncertainty research of water resource complex problems. However, in the rapid evolution of the adjoint function, some problems greatly limit the application of the adjoint function in the research of water resources. Therefore, based on bibliometric analysis, development, practical application issues, and prospects of the hot directions are analyzed. It is found that the development of the connection number of water resource set pair analysis can be divided into three stages: (1) relatively sluggish development before 2005, (2) a period of rapid advancement in adjoint function research spanning from 2005 to 2017, and (3) a subsequent surge post-2018. The introduction of the adjoint function of connection number promotes the continuous development of set pair analysis of water resources. Set pair potential and partial connection number are the crucial research directions of the adjoint function. Subtractive set pair potential has rapidly developed into a relatively independent and important trajectory. The research on connection entropy is comparatively less, which needs to be further strengthened, while that on adjacent connection number is even less. The adjoint function of set pair potential can be divided into three major categories: division set pair potential, exponential set pair potential, and subtraction set pair potential. The subtraction set pair potential, which retains the original dimension and quantity variation range of the connection number, is widely used in water resources and other fields. Coupled with the partial connection number, a series of new connection number adjoint functions have been developed. The partial connection number can be mainly divided into two categories: total partial connection number, and semi-partial connection number. Among these, the calculation expression and connotation of total partial connection numbers have not yet reached a consensus, accompanied by the slow development of high-order partial connection numbers. Semi-partial connection number can describe the mutual migration movement between different components of the connection number, which develops rapidly. With the limitations and current situation described above, promoting the exploration and application of the adjoint function of connection number in the field of water resources and other fields of complex systems has become the focus of future research.

## 1. Introduction

Water resources are key elements and important support for human survival and for promoting the sustainable development of social economy. Affected by climate change and human activities, water shortage, water ecological environment deterioration, and water disasters have become the main bottlenecks restricting the sustainable development of the economy and society [1]. The water resource system is a complex system formed by the interaction between the development, utilization, and protection of water resources and multi-factors of population, economy, social activities, and ecological environment. With the development of economy and increased population, the demand for water resources keeps increasing, exacerbating the waste and pollution of water resources. Global water system security is facing increasingly serious challenges [2]. Water security, such as analysis, evaluation, prediction, and decision-making of water resource complex systems, has been increasingly emphasized by scholars [3,4]. Various complex systems analysis methods, such as probabilistic statistics, fuzzy mathematics, grey system, optimal decision-making, etc., have been proposed and applied in the research of water resources systems. However, the stochastic nature of the hydrological cycle, the ambiguity of flood season, the complexity of water resources transformation, and the uncertainty of water resources protection have greatly increased the uncertainty of water resource complex system [5].

Keqin Zhao, a Chinese scholar, believed that the complexity of system uncertainty should be studied by adopting the research idea of system science and dialectical thinking, and proposed the system uncertainty analysis theory called Set Pair Analysis (SPA) in 1989 to deal with the quantitative analysis of deterministic uncertainty systems [6]. SPA is one of the important artificial intelligence theories [7,8], the core idea of which is based on the principle of universal connection and the pairwise existence of things. SPA regards certainty and uncertainty as a system and constructs the connection number from three aspects (sameness, differences, and opposites) to quantitatively analyze the relationship structure between certainty and uncertainty and their mutual transformation. SPA lays a foundation for the construction of connection science [9,10].

As a fundamental tool of set pair analysis, the connection number serves as a structural function to study the quantitative measurement of the state of uncertainty in dialectical relationships between two set pair events. The connection number can portray the degree of set pair relationship from three aspects: sameness, differences, and opposites, which can quantitatively characterize the multi-level deterministic uncertainty characteristic relationship in the system. Different definitions and operations of the connection components and difference coefficients can be used to generate adjoint functions such as set pair potential, partial connection number, and connection entropy of the connection number, which can further reveal the evolution characteristics of the various deterministic-uncertainty relationships of the uncertainty events and greatly expand the research and application fields of connection numbers [11,12].

A majority of practice in water resources management showed the necessity to deal with the complex relationship between various elements in the complicated system of water resources, water disasters, water environment, water ecological protection, and management. These relationships are interrelated, opposite, and unified, and constitute a variety of set pairs in the phenomenon of water resources, which provides a wide range of application basis for the set pair analysis of water resources [13]. The set pair analysis of water resources is to construct two relationship sets based on the identification of water resources problems, and comprehensively express its attribute relationship from the aspects of sameness, difference, and opposites through connection number and its adjoint function [14,15]. SPA is similar to the fuzzy hydrology and stochastic hydrology methods, which consider both the quantitative computation of hydrological water resources problems and the physical analysis of the computational process that is typical computational thinking [16]. Compared with traditional methods, the set pair analysis method of water resources is more intuitive, concise, and comprehensive when studying of water resource complex problem, and has been rapidly applied to the research field of analysis, evaluation, prediction, and decision-making of the water resources complex system [17,18,19,20,21].

The construction of the adjoint function of connection number is the hot spot and frontier of application in the field of current connection number research [13]. To date, a variety of adjoint functions have been proposed, such as set pair potential [8,22], partial connection number [23,24], adjacent connection number [25,26], connection entropy [27,28], and so on. In recent years, the adjoint functions, such as subtraction set pair potential, total partial connection number, semi-partial connection number, and coupled adjoint function, developed rapidly, especially in the fields of water resources carrying capacity evaluation, flood and drought risk assessment, water resources security, and runoff classification and prediction.

However, with the advancement of the adjoint functions of connection number, there are some problems, including inconsistency in the concept of adjoint functions, unclear physical connotations in the calculation process, inconsistent formulas, and even errors, greatly limiting further progress of the adjoint function. Meanwhile, the history of the adjoint function of connection number is comparatively short, only around 20 years, and even more recently when applied to the field of water resource analysis, which makes the fundamental theory and practical application of the current adjoint function of connection number relatively weak. Overall, the application of the adjoint function of connection number requires continued expansion and exploration [13,29].

To further sort out the development process of the adjoint functions and promote the investigation and application of the adjoint function of connection number in the water resources system, this paper conducted a bibliometric analysis of the adjoint functions of the set pair analysis of water resources. The development process, research field, and practical application of the set pair analysis of water resources were examined. In this paper, the researchers focused on the research status, existing problems, and development trends of the hot topics such as determination method of the sameness–difference–opposition relationship, set pair potential, total partial connection number, semi-partial connection number, and coupled adjoint functions. The problems were put forward to advance the future development of the adjoint function of water resource set pair analysis.

## 2. Basic Concepts of Adjoint Functions of Connection Number in SPA

### 2.1. Connection Number

Set pair analysis is a kind of uncertainty analysis theory. It originates from the dialectical thinking that the world is the unity of opposites between certainty and uncertainty. It regards certainty and uncertainty as a complete certainty–uncertainty system, and uses the connection number (Equation (1)) to portray the unity of opposites between certainty and uncertainty of the system from three aspects: sameness, difference, and opposites [9,30].

(1) u=a+bi+cj

where *u* is the connection number, *a* is the degree of congruence, *b* is the degree of difference, *c* is the degree of opposites, *i* is the coefficient of difference, and *j* is the coefficient of opposites, satisfying:

(2) $$\left\{ \begin{array}{l} a+b+c=1,\\ a,\;b,\;c\in [0,\;1],\\ i\in [-1,\;1],\\ j=-1 \end{array}\right.$$

Correspondingly, according to Equations (1) and (2), the SPA method can be extended to multivariate connection number [14].

(3) $$\left\{ \begin{array}{l} u=a+b_{1}i_{1}+b_{2}i_{2}+\cdots +b_{n}i_{n}+cj,\\ a+b_{1}+b_{2}+\cdots +b_{n}+c=1,\\ a,\;b_{1},\;b_{2},\;\cdots ,\;b_{n},\;c\in [0,\;1],\\ i_{p}\in [-1+\frac{2(p-1)}{n},-1+\frac{2p}{n}],p=1,\;2,\;\cdots ,\;n,\\ j=-1 \end{array}\right.$$

where *b*_1_, *b*_2_, …, *b_n_* represent the expansion of *b_i_*, and *n* is the number of *b*.

The connection number is a structural function used to describe certainty and uncertainty, and the interaction between certainty and uncertainty in the set pair events studied. It is a quantitative expression of the same, different, and opposite relationship between two set pair events in a certain problem [6,9,13].

Among them, the sameness degree item *a* and the opposites degree item *c* are relatively certain fuzzy relations, and the difference degree item *b* is a relatively uncertain fuzzy relation. At the same time, the same degree, the different degree, and the opposite degree are determined at the macro level, while the difference degree coefficient is uncertain at the micro level and can continue to be decomposed. The uncertainty of the problem can be adapted by dynamically adjusting the value of the different degree coefficient, and the deterministic uncertainty of the system can be reflected by the aspects of sameness, difference, and opposites.

### 2.2. Basic Concept of Adjoint Function

The adjoint function of connection number is a function that represents the relationship structure of the event at the macro and micro levels, which is composed of the connection components *a*, *b*, and *c*, the difference coefficient *i*, and the opposition coefficient *j* in the connection number [9].

The connection components *a*, *b*, and *c* represent the uncertainty of the event at the macro level, and the difference coefficient *i* and the opposition coefficient *j* represent the uncertainty of the event at the micro level. In the case of the connection components *a*, *b*, and *c* of the connection number given, the construction of the structure-function based on *a*, *b*, and *c*, and the difference coefficient *i*, can comprehensively and deeply reflect the transformation between the connection components and the development trend of the set pair events [11]. At present, there are a variety of adjoint functions: set pair potential, partial connection number, semi-partial connection number, adjacent connection number, connection entropy, etc. Among them, set pair potential and partial connection number are the most widely developed and applied.

#### 2.2.1. Set Pair Potential

In the expression of connection number (1), the connection components *a*, *b*, and *c* can, respectively, reflect the relationship level of set pair events in the same, different, and opposite directions. At the same time, the relative size difference between the three can further express the development trend of set pair events to a certain extent. Therefore, in 2000, Zhao Keqin proposed the set pair potential to characterize this connection trend, which is calculated as follows [9]:

(4) shi(H)=a/c

where *shi*(*H*) represents the set pair potential; when *shi*(*H*) > 1, it is the same potential; *shi*(*H*) = 1 is the equilibrium, *shi*(*H*) < 1 is the anti-potential, and further combined with the size of *b*, different levels and trend relationships with more detailed set pair potential are divided.

#### 2.2.2. Partial Connection Number

The connection number belongs to the relational grade function. As a quantitative calculation of the change rate from one grade to the adjacent grade, Keqin Zhao proposed the concept of partial connection number in 2005 [23]: from the perspective of development, it is assumed that the current state of “the same” was also at the level of “different”, which is developed from the relatively indeterminate “different”. So, the degree of the development can be expressed as *a*/(*a* + *b*), denoted ∂a = a/(*a* + *b*). Similarly, it is assumed that the current state of “different” was originally at the level of “opposite”, which is developed from the relatively determined “opposite”. The degree of this development can be expressed as *b*/(*b* + *c*), denoted as ∂*b* = *b*/(*b* + *c*).

Let the partial positive connection number ∂^+^*µ* = ∂*a* + *i*∂*b* for the connection number *a* + *bi* + *cj*, and *i* = ∂*a*/(∂*a* + ∂*b*) according to the principle of proportionality. Similar to the partial negative connection number ∂^−^*µ* = *i*∂^−^*b* + ∂^−^*c*, from which the partial connection number can be obtained as follows [31].

partial positive connection number:

(5) $$\partial^{+}\mu =\frac{a}{a+b}+\frac{b}{b+c}i_{1},i_{1}\in [-1,\;1]$$

partial negative connection number:

(6) $$\partial^{-}\mu =\frac{b}{a+b}i_{2}+\frac{c}{b+c}j,i_{2}\in [-1,\;1],j=-1$$

total partial connection number:

(7) $$\partial \mu =\partial^{+}\mu -\partial^{-}\mu =\frac{a}{a+b}+\frac{b}{b+c}i_{1}+(\frac{b}{a+b}i_{2}+\frac{c}{b+c}j),i_{1},i_{2}\in [-1,\;1],\;j=-1$$

where ∂^+^*μ* reflects the positive development trend of the research object, ∂^−^*μ* reflects the negative development trend of the research object, ∂*μ* represents the comprehensive development trend of the research object, *i*_1_, *i*_2_ for the uncertainty coefficient, and *j* for the indicative coefficient, generally taken as −1. The partial connection number describes the dialectical transformation between the same, different, and opposite relations of the same set pair relationship degree, rather than the system hierarchical transformation relationship. The same, different, and opposite relations are the division of different classes of the connection degree of the set pair system. The three belong to the same level of set pair relationship degree, and the same, different, and opposite relations have different dimensions. In general, the partial connection number can be used to characterize the change of the uncertainty state in the set pair event and judge the development trend of the event.

## 3. Application and Development of Set Pair Analysis in Water Resource Complex System

The Chinese Academic Journals Network Publishing Database, Chinese Important Conference Papers Database, Chinese Doctoral Dissertations Database, and Chinese Outstanding Master’s Degree Dissertations Database of China Academic Journals Full Text Database (CNKI) were selected for the advanced search, with the common subject terms of ‘of pairs’ and ‘water resources’. The period was from 1989 to 2022, and a total of 496 related documents were obtained, but the earliest document retrieved was from 2001, setting the period of statistical analysis as 2001–2022. The Web of Science Core Collection (WOS) database was selected, and the literature published from 2003 to 2022 was searched with ‘Topic = (set pair analysis AND water resources)’. A total of 112 search results were obtained. Bibliometric analysis was used to analyze the publication time, disciplines, and keywords of the above retrieved literature to reveal the overall research progress and potential problems of water resource set pair analysis.

### 3.1. Analysis of the Development Time of Water Resource Set Pair Analysis

**Figure 1:**
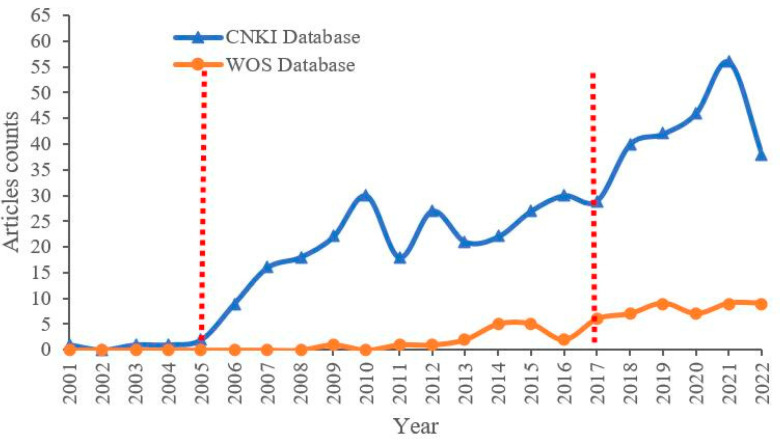
Number of papers published on water resource set pair analysis by years.

As shown in Figure 1, since 2001, the overall development of water resource set pair analysis research papers generally shows a trend of fluctuating growth. The number of Chinese papers in the same period is significantly higher than that from other countries, and the research results of set pair analysis of water resources have great potential to be promoted to other countries. The CNKI library shows that, in 2001, Wang Dong attempted to apply the fundamental concepts and theories of set pair analysis (SPA) and fuzzy set theory (FST) to establish a water environment evaluation model based on SPA and SPA-FST [32]. Before 2005, the development of water resource set pair analysis research was slow, while the number of papers issued from 2006 to 2010 increased rapidly, followed by an obvious surge in 2010, with more than 30 papers issued in one year, which is mainly in the comprehensive evaluation of water resources. The fluctuating growth trend was observed from 2011 to 2022, where three obvious rapid growth periods showed up in 2012, 2018 and 2021, respectively, with the highest number of articles in 2021, reaching 56 articles. It can be foreseen that water resource set pair analysis research is becoming a rapidly developing research field.

In the WOS library, set pair analysis was applied to water resource analysis by Wang Wensheng in 2009 [15] for the evaluation and analysis of water resource system. The number of papers published in the WOS database is significantly lower than that in the CNKI database, implying that set pair analysis mainly focuses on the research of Chinese scholars. The set pair analysis method is a quantitative expression of the unity of opposites in dialectics proposed by Chinese scholars, which is different from the qualitative description of the unity of opposites in traditional philosophy. Although it is developing rapidly in recent years, it has only around 30 years of development history since it was proposed, and only around 20 years of applied research in water resources, making the fundamental theory and application relatively weak. Most of the research on set pair analysis has been published in Chinese, obstructing the recognition for international scholars who are not native speakers of Chinese. As a relatively new method, set pair analysis may not yet be widely accepted and appreciated by the international academic community. International scholars may prefer to use traditional methods that have been widely validated and recognized, such as probability statistics and fuzzy mathematics. The international academic conferences, journals, and other platforms have less introduction and discussion of the set pair analysis method, limiting the opportunities for international scholars to access and use this method. However, the papers published in the WOS database show an increasing trend year by year. The water resource set pair analysis has great potential to be promoted to other countries.

From the process of the application of set pair analysis theory and method in water resource system analysis, before 2005, the fundamental theory of set pair analysis was mainly introduced into water resources system analysis to study the water resources set pair system from the macroscopic static aspect. From 2005 to 2017, the dynamic partial connection number of micro-analyses was gradually introduced into the study of the water resources system, which greatly promoted the rapid development of water resource set pair analysis. The application of adjoint function methods, such as the subtraction set pair potential in 2018 and the semi-partial connection number in 2020, in the application of water resources issues has promoted the research and application of water resource set pair analysis methods into a new stage of high-speed development, and the number of publications reached a peak in 2021. It is suggested that the overall development trend of water resource set pair analysis is closely related to the development of new adjoint functions of set pair analysis.

### 3.2. Discipline Analysis of Water Resource Set Pair Analysis

**Figure 2:**
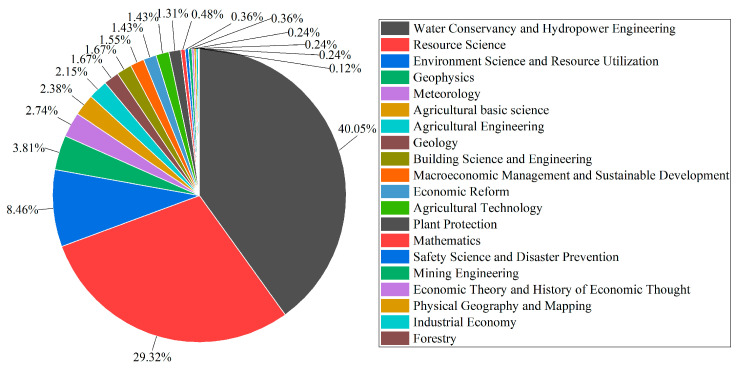
The proportion of top 20 academic disciplines for water resources SPA papers in CNKI (2001–2022).

In Figure 2, it is demonstrated that the main disciplines in the field of water resource set pair analysis are water conservancy hydropower engineering and resource science. The number of publications is more than 200, accounting for 40.05% and 29.32%, respectively, the cumulative proportion of which is close to 70%, followed by environmental sciences and resource utilization, with a share of 8.46%, and geophysics, meteorology, basic agricultural sciences, agricultural engineering, and other disciplines that account for less than 5% of the total number of articles. It suggested that water conservancy hydropower engineering and resource science are strong disciplines in the field of set pair analysis of water resources. The set pair analysis of water resources in the environment, geology, meteorology, agriculture, and other related disciplines also showed gradually expanding.

### 3.3. Keyword Analysis of Water Resource Set Pair Analysis Studies

**Figure 3:**
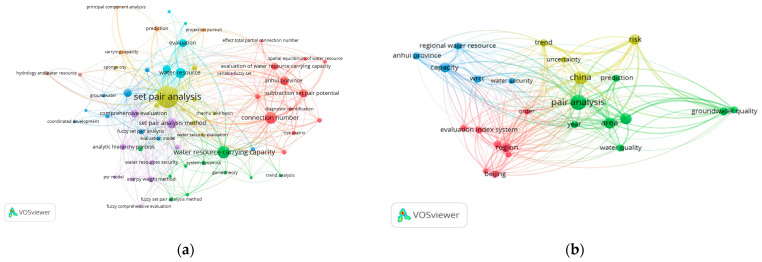
Keyword co-occurrence labelling map for water resource set pair analysis, where (**a**) keyword co-occurrence labelling map for water resource set pair analysis in CNKI database; (**b**) keyword co-occurrence labelling map for water resource set pair analysis in WOS database.

**Figure 4:**
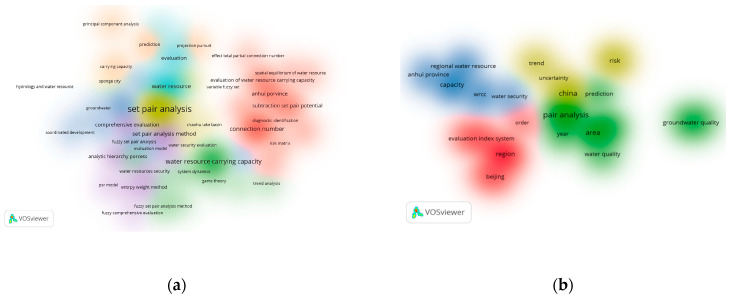
Keywords co-occurrence clustering density map for water resource set pair analysis, where (**a**) keywords co-occurrence clustering density map for water resource set pair analysis in CNKI database; (**b**) keywords co-occurrence clustering density map for water resource set pair analysis in WOS database.

The VOSviewer 1.6.19 tool [33] was used to conduct a bibliometric analysis of 496 papers in the CNKI database and 112 papers in the WOS data on water resource set pair analysis. The network visualization analysis diagram (Figure 3) and density visualization analysis diagram (Figure 4) of the keywords of water resource set pair analysis were generated.

Each point in Figure 3 represents the core word of the paper. The larger the dot is the greater the number of papers related to that core word. In the CNKI database, water resource set pair analysis research can be divided into four clusters: the first category is mainly applied to the evaluation of water resources carrying capacity, and the main methods such as connection number, partial connection number, and set pair potential are widely used. Anhui Province and Shandong Province are the main research areas for this category. The second category mainly includes water resources vulnerability assessment, water security assessment, and sustainable use, where set pair analysis is applied in combination with the entropy weight method, fuzzy analysis, system dynamics, and other methods. The third category is mainly water quality evaluation, groundwater evaluation, coordinated development evaluation, and other research. The fourth category focuses on the evaluation and prediction of the uncertainty of the hydrological water resources system using the connection degree and projection tracing methods. In the WOS database, there are four main categories, similar to CNKI, mainly including WRCC, capacity, water security; water quality, groundwater quality; evaluation index system; risk, trend, uncertainty, and other four categories of keywords for comprehensive evaluation and prediction of water resources system.

The density visualization analysis diagram (Figure 4), suggested that the set pair analysis of water resources can be relatively clear in CNKI and WOS data, with about four density centers, respectively, which is consistent with the cluster of Figure 3. However, the clustering density map of the CNKI database suggested that some keywords penetrate, cross, or even overlap between various categories. The keyword of evaluation appears in all five categories. The fuzzy relationship penetrates four clusters, and the main research topics such as carrying capacity and water safety cross appear. It can be seen that evaluation is the most widely used set pair analysis in the field of water resources research, and the analysis and processing of the same–difference–opposite fuzzy relationship in the set pair analysis of water resources showed an important correlation of the research.

### 3.4. Main Applications of Water Resource Set-Pair Analyses

**Figure 5:**
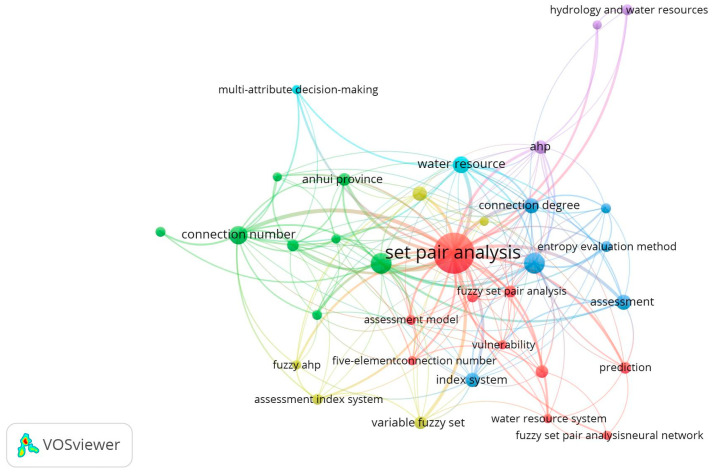
Co-occurrence labelling map of keywords for the main applications of water resources set-pair analyses.

The application of the set pair analysis method in water resources mainly includes water resources analysis, evaluation, prediction, and decision-making. In the CNKI database, ‘water resources’, ‘set pair analysis’ + ‘prediction’, ‘evaluation’, and ‘decision’ were used as subject words for advanced search, and VOSviewer 1.6.19 software was used for keyword analysis to generate keyword co-occurrence label map (Figure 5).

As shown in Figure 5, in terms of the application of water resource set pair analysis, the core word ‘evaluation’ appears most frequently and is most closely related to each other, which is mainly applied in the evaluation of water resource carrying capacity, water security, vulnerability assessment, and other fields. The methods of connection number, set pair potential, fuzzy analysis, and entropy are widely used. In terms of water resource prediction, the core word of ‘prediction’ appears, mainly for water resources, precipitation, runoff, and other aspects of individual prediction. In terms of water resource decision-making, the ‘multi-attribute decision-making’ core word shows up with a relatively small degree of linkage with other core words, which still belong to the category of comprehensive evaluation, and makes decisions through the evaluation of different schemes. It is suggested that integrated evaluation is the main application aspect of water resource set pair analysis.

**Figure 6:**
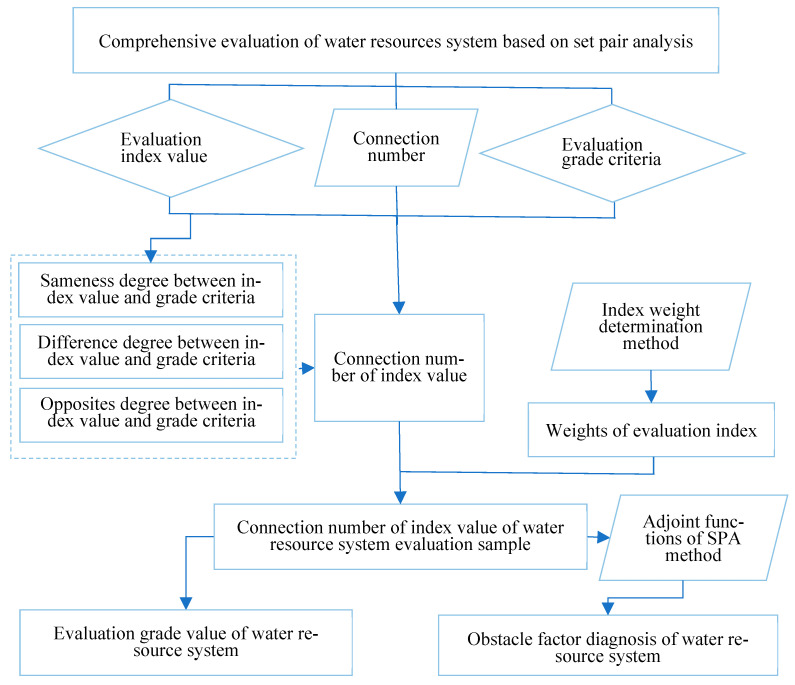
Set pair evaluation framework for water resources system.

Taking the most widely used set pair evaluation of water resources as an example, a framework diagram (Figure 6) is drawn to illustrate its specific application.

Figure 6 demonstrated that in the comprehensive evaluation of water resources, the set pair analysis method comprehensively evaluates the uncertainty of a single index from three aspects: same degree, difference degree, and opposition degree, which is significantly better than the traditional fuzzy membership degree, grey correlation degree, and correlation coefficient that can only be evaluated from a single perspective. Meanwhile, the use of the connection number adjoint function can further diagnose and analyze the obstacle factors of the evaluation results, providing a reference for decision-making.

In addition to comprehensive evaluation, set pair analysis extensively analyzes the certainty and uncertainty of water resource set pair from the same, different, and opposite aspects, and is also broadly used in the field of water resource analysis, prediction, and decision-making. Compared with the traditional methods, it highlights the benefits of a simple and clear model, comprehensive analysis, good prediction accuracy, and reasonable decision-making. For example, Zhu et al. (2007) [34] compared the connection degree, correlation coefficient, fuzzy membership degree, and grey correlation degree in the analysis of the relationship between flood peaks and flows. They found that the correlation of hydrological variables reflected by each index was consistent, but the set pair analysis connection degree described the relationship structure between hydrological and water resources variables more completely. Mo et al. (2021) [35] used the set pair analysis similar prediction model to predict the runoff in the typical karst area of southwest China, which proves that it has good applicability in the prediction of medium- and long-term water resources in the basin with insufficient measured data. Wu et al. (2016) [36] used set-pair analysis to make decisions on water resource allocation schemes, and the results were in good agreement with the fuzzy preference method, fuzzy entropy model, neural network model, and lattice order theory model. The application of set pair analysis in the uncertainty analysis of water resource complex system is more and more extensive.

## 4. Development of Adjoint Function for Set Pair Analysis in Water Resources Complex Systems

Constructing the adjoint function based on the variables such as the connection number components *a*, *b*, and *c*, the difference coefficient *I* and the opposition coefficient *j* can further decompose the macro-level research into the micro-level analysis in different directions, and then quantitatively describe the set pair from the multi-level and multi-scale. Adjoint functions can quantitatively express the interrelationships, interactions, and evolutionary trends between things, and provide a novel exploration way for dealing with complex uncertainty problems.

**Figure 7:**
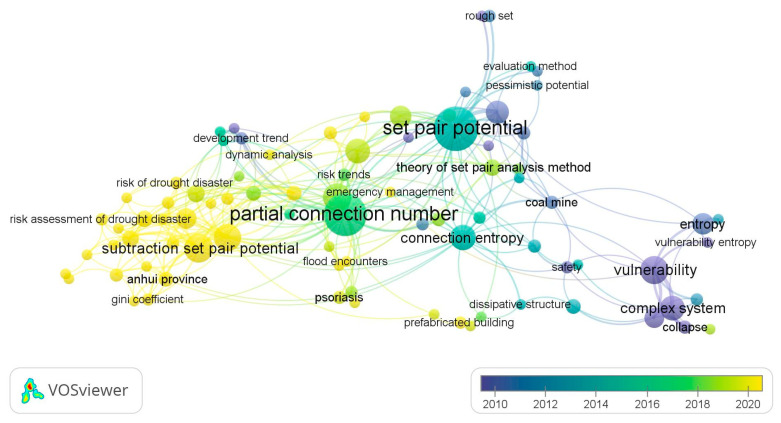
Co-occurrence labelling map of keywords for the study of the main conjunct function of connection number.

In the CNK database, ‘set pair potential’, ‘partial connection number’, ‘adjacent connection number’, and ‘connection entropy’ were used as keywords for advanced search, and VOSviewer 1.6.19 software was used to analyze the keywords of adjoint function. To show the research hotspots of the concomitant function more clearly, the keywords of ‘set-pair analysis’ and ‘connection number’ with too high frequency were not shown, and the keyword co-occurrence labelling map of the adjoint function research was generated (Figure 7).

It can be seen from Figure 7 that there are many studies on “set pair potential “and “partial connection number” in the adjoint function of connection number, which is widely used in the field of water resources carrying capacity, risk assessment, hazard evaluation, complex system analysis, and so on. In recent years, the ‘subtraction set pair potential’ [21] has developed rapidly into a relatively independent research direction [21] and developed the other coupled adjoint function research branches such as the ‘semi-subtraction set pair potential’ [37], which are validated by stochastic simulation method [38]. The adjoint functions are mainly applied to the evaluation of water resources carrying capacity [39,40], drought risk assessment [37,41], and other research fields. Meanwhile, the adjoint functions are widely used in combination with the multivariate (three-element, five-element) connection number. However, the research on connection entropy is relatively less [27,42], and most of the related research mainly focuses on the combination of set pair analysis and entropy weight [43,44,45], implying the research on connection entropy needs to be further strengthened. Furthermore, the study of adjacent connection numbers is even less.

It also suggested that set pair potential and partial connection number are the most important research directions of the current connection number adjoint function. The coupling of set pair potential and partial connection number into a new adjoint function will be an important development direction in the future. The adjoint function of connection number is widely used in the field of water resources. In the future, it can be expanded into other key research directions such as risk assessment and risk assessment of complex systems.

Set pair is the cornerstone of describing the relationship between certainty and uncertainty of the research objects, in which the quantitative determination of the same–different–opposite relationship is the basis for the calculation of the component of the connection number. The adjoint function can further explore the mutual transformation of the research objects at the micro level. Therefore, combined with the main development direction of the above-mentioned connection number adjoint function, the following focuses on the analysis of the adjoint function of water resource set pair analysis from the perspectives of the determination of the same, different, and opposite relationship of the connection number, the set pair potential, the partial connection number, the coupling adjoint function, and other opposite connection number adjoint functions.

### 4.1. Methods for the Determination of the Same-Different-Opposite Relationship

In the field of water resource set pair analysis, the methods of the determination of the same–different–opposite relationship can be summarized into the following three categories.

#### 4.1.1. Quantitative Characterization of Connection Number by Segmental Functions of Set Pair Variables

**Figure 8:**
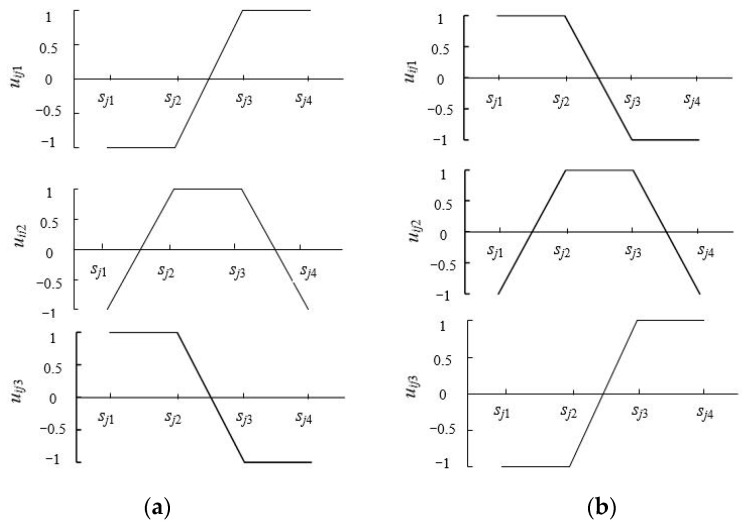
Schematic diagram of the connection number piecewise function. (**a**) Positive indicators, (**b**) negative indicators.

The evaluation sample index and its corresponding evaluation standard are taken as two sets, respectively, which constitute a set pair. If the sample index value is in a certain evaluation level, it is regarded as the same as the level, and the connection number is 1. If the index value falls into the interval grade, it is regarded as the opposite, and the connection number is −1; if the index value falls into the adjacent level, it is regarded as different and the difference coefficient *i* changes in the interval of [−1, 1]. The closer to the same level, the closer to 1, the closer to the inverse level, and the closer to −1. The corresponding segmental function of the connection number of the single index evaluation in the commonly used three-element connection number can be constructed as follows [22,46]:

Set pair analysis is used to construct the single index connection number *u_ijk_* (−1 ≤ *u_ijk_* ≤ 1) between the sample value *x_ij_* of the water resources complex system sample *i* index *j* and the evaluation grade standard s*_jk_*. If the sample value *x_ij_* increases with the increase of the evaluation grade (positive index) or decreases with the increase of the evaluation grade (negative index), taking the evaluation system of each indicator divided into 3 levels as an example, the single index evaluation connection number *u_ijk_* is calculated as follows [22,46]:

(8) $$u_{ij1}=\{\begin{array}{l} 1,\mathrm{positive}\;\mathrm{index}\;x_{ij}\leq s_{j1},\mathrm{or}\mathrm{negative}\;\mathrm{index}\;x_{ij}\geq s_{j1};\\ 1-2(x_{ij}-s_{j1})/(s_{j2}-s_{j1}),\mathrm{positive}\;\mathrm{index}\;s_{j1}<x_{ij}\leq s_{j2}\;\mathrm{or}\mathrm{negative}\;\mathrm{index}\;s_{1jk}>x_{ij}\geq s_{j2};\\ -1,\mathrm{positive}\;\mathrm{index}\;x_{ij}>s_{j2}\mathrm{or}\mathrm{negative}\;\mathrm{index}\;x_{ij}<s_{j2} \end{array}$$

(9) $$u_{ij2}=\left\{ \begin{array}{l} 1-2(s_{j1}-x_{ij})/(s_{j1}-s_{j0}),\mathrm{positive}\;\mathrm{index}\;s_{0jk}<x_{ij}\leq s_{j1}\;\mathrm{or}\mathrm{negative}\;\mathrm{index}\;s_{0jk}>x_{ij}\geq s_{j1};\\ 1,\mathrm{positive}\;\mathrm{index}\;s_{j1}<x_{ij}\leq s_{j2}\;\mathrm{or}\;\mathrm{negative}\;\mathrm{index}\;s_{j1}>x_{ij}\geq s_{j2};\\ 1-2(x_{ij}-s_{j2})/(s_{j3}-s_{j2}),\mathrm{positive}\;\mathrm{index}\;s_{j2}<x_{ij}\leq s_{j3}\;\mathrm{or}\mathrm{negative}\;\mathrm{index}\;s_{j2}>x_{ij}\geq s_{j3};\\ -1,\mathrm{positive}\;\mathrm{index}\;x_{ij}>s_{j3}\;\mathrm{or}\mathrm{negative}\;\mathrm{index}\;x_{ij}<s_{j3} \end{array}\right.$$

(10) $$u_{ij3}=\{\begin{array}{l} -1,\mathrm{positive}\;\mathrm{index}\;x_{ij}\leq s_{j1}\;\mathrm{or}\;\mathrm{negative}\;\mathrm{index}\;x_{ij}\geq s_{j1};\\ 1-2(s_{j2}-x_{ij})/(s_{j2}-s_{j1}),\mathrm{positive}\;\mathrm{index}\;s_{j1}<x_{ij}\leq s_{j2}\;\mathrm{or}\;\mathrm{negative}\;\mathrm{index}\;s_{j1}>x_{ij}\geq s_{j2};\\ 1,\mathrm{positive}\;\mathrm{index}\;x_{ij}>s_{j2}\;\mathrm{or}\mathrm{negative}\;\mathrm{index}\;x_{ij}<s_{j2} \end{array}$$

where *s_jk_* are the critical values of the *k*th level of index *j*. From the theory of set pair analysis, it is known that if the connection number between the sample and a certain evaluation grade is the largest, the sample is considered to belong to this grade [22] (Figure 8).

In the five-element or more multivariate element connection numbers, when the sample value *x_ij_* is the same grade as the evaluation standard *s_jk_*, the connection number *u_ijk_* = 1; adjacent grade, *u_ijk_* ∈ [−1, 1], and *u_ijk_* = −1 when *x_ij_* and *s_jk_* are separated by a grade. In this way, when the calculated single-index connection number is converted into membership degree and calculation connection component, there will be multiple zero value problems. It can be solved by taking two or more grades as the opposite, shortening the opposition interval of the connection number, to slow down the slope of the two adjacent grades in the connection number segmental function. At the same time, it can further enrich the physical meaning of the single index connection number between the sample value and the evaluation standard grade [37].

#### 4.1.2. Quantitative Characterization of Connection Number by Hierarchical Classification of Set Pair Variables

The set pair variables of water resources and their influencing factors are classified in the range of variation, and the set pair analysis is carried out with the connection number of the corresponding elements. The common three-level and five-level classification methods are as follows:

(1) Three-level classification method: the water resource variable *y_i_* and the influence factor variable *x_ij_* are symbolized by mean plus or minus 0.44 times the standard deviation using the mean standard deviation method [17]:

(11) $$z_{ij}=\{\begin{array}{l} \begin{array}{cc} 1, & x_{ij}\leq {\bar{x}}_{j}-0.44s_{j} \end{array}\\ \begin{array}{cc} 2, & {\bar{x}}_{j}-0.44s_{j}<x_{ij}<{\bar{x}}_{j}+0.44s_{j} \end{array}\\ \begin{array}{cc} 3, & x_{ij}\geq {\bar{x}}_{j}+0.44s_{j} \end{array} \end{array}$$

(12) $$z_{i0}=\{\begin{array}{l} \begin{array}{cc} 1, & y_{i}\leq \bar{y}-0.44s_{0} \end{array}\\ \begin{array}{cc} 2, & \bar{y}-0.44s_{0}<y_{i}<\bar{y}+0.44s_{0} \end{array}\\ \begin{array}{cc} 3, & y_{i}\geq \bar{y}+0.44s_{0} \end{array} \end{array}$$

where ${\bar{x}}_{j}$ and $s_{j}$ are the mean and standard deviation of *x_ij_*, respectively; ${\bar{y}}_{i}$ and $s_{0}$ are the mean and standard deviation of *y_j_*, respectively; *z_ij_*, *x_i_*_0_ are the graded quantities of *x_ij_* and *y_j_*, respectively. The connection number is defined by comparing the corresponding components of the set pair *H* (*z_i_*_0_, *z_ij_*). If the corresponding components are at the same level, adjacent level, and separated level, respectively, they represent the sameness, difference and opposites of the set pair system. The three-element connection number can be constructed to express the relationship between water resource variables and their influencing factors.

(2) Five-level classification method:

According to the change characteristics of water resources variable *y_i_*, such as runoff can be divided into five levels: special low (I), low (II), medium (III), high (IV), and special high (V), and the mean standard deviation method was used to classify the flow by the intervals of [0, −0.9σ], [−0.9σ, −0.3σ], [−0.3σ, +0.3], [+0.3σ, +0.9σ], and [+0.9σ, +∞], respectively [47], where ${\bar{y}}_{i}$ and σ are the mean and variance of the mean flow of the water resource variable *y_i_*, respectively. The water resource sample value falls in a certain level, denoted as the same, with a difference of one level as the difference one, recorded as *b*_1_, with a difference of two levels as the difference two, recorded as *b*_2_, and with a difference of three levels and above is the opposite, recorded as the opposite, so that the quaternary connection number can be constructed to express the variation characteristics of water resources variables.

Similarly, combined with the physical meaning of water resources variables, different quantitative classifications can be carried out within the range of the variable changes, and combined with the connection number for hierarchical symbolization, the connection number of the corresponding elements can be constructed, to comprehensively analyze the relationship between water resources variables and related factors from the perspective of the same, different, and opposite.

#### 4.1.3. Quantitative Characterization of Connection Number by the Interactions of Set Pair Variables

A certain interaction relationship between the water resources variable *y_i_* and its related variable *x_j_* is constructed as an evaluation index *z_ij_*. The index and its corresponding evaluation criteria are used as two sets to form a set pair. The sample index values are in a certain evaluation level, falling in the adjacent level, and the interval level is defined as the same, different, and opposite for quantitative calculation. For example, the total amount of water resources *y_i_* is the matching image, and the variable *x_j_* (cultivated land area *x*_1_, population *x*_2_, and secondary industry GDP *x*_3_) is the matching object of water resources. Based on the Gini coefficient, the matching relationship between the matching image and the matching object *Z_ij_* (*y*-*x*_1_, *y*-*x*_2_, *y*-*x*_3_) is used as the evaluation index, and the set pair of *Z_ij_* and Gini coefficient level is constructed to analyze the matching equilibrium degree between the total amount of water resources and each index [48].

Considering the methods described above, the relationship variables between different water resources indicators and the indicators with interaction relationship can be constructed, the relationship between the variable and its reference-grade standard can be calculated, and the interaction relationship between water resources variables and other variables can be analyzed.

It suggested that the connection number can be constructed from different points of view, such as constructing the segmental function of water resources variables and reference criterion, quantifying the level of water resources variables, and constructing the relationship between water resources variables and their related variables, to characterize the connection degree of the same-different-opposite in water resource set pair analysis.

### 4.2. Set Pair Potential Method

**Figure 9:**
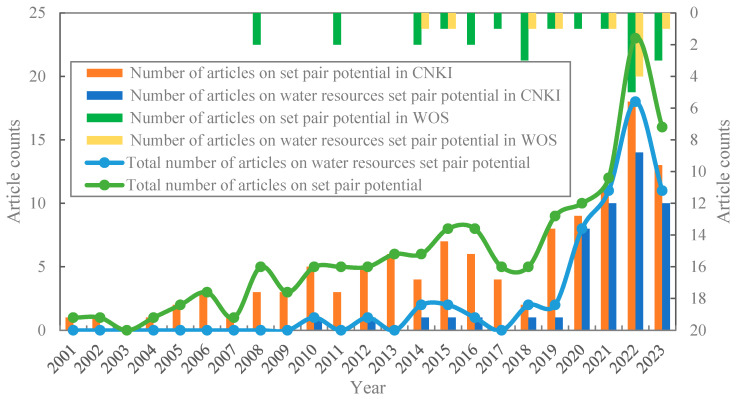
Statistics of the number of papers published in CNKI and WOS.

**Figure 10:**
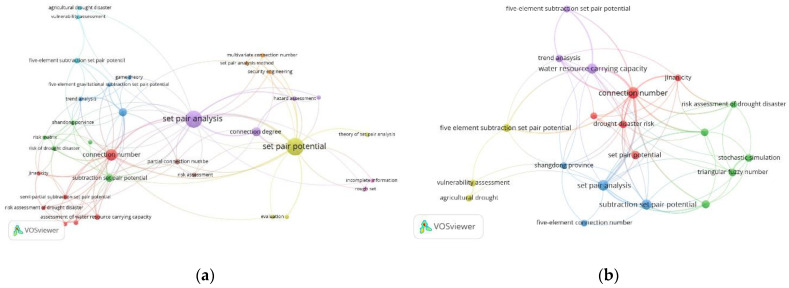
Keyword co-occurrence labelling map for set pair potential, where (**a**) keyword co-occurrence labelling map of set pair potential in CNKI database; (**b**) keyword co-occurrence labelling map for water resource set pair potential in CNKI database.

The connection number components *a*, *b*, and *c* in the set pair analysis reflect the degree of the same, different, and opposite relationship between the set pair events, and the size difference between the three expresses the development trend of the set pair events under a certain problem to a certain extent [9]. In CNKI, an advanced search was conducted with “set pair potential” as the theme, and up to October 2023 there were 118 related papers, from which a total of 49 papers were screened for hydrological water resources-related research. In the WOS, a total of 23 articles were retrieved with the theme of “set pair potential” or “set pair connection potential”, and 10 studies related to water resources were selected for statistical analysis (Figure 9). The VOSviewer 1.6.19 tool was used to analyze and generate a set pair potential keyword network visualization analysis diagram (Figure 10).

It can be seen from Figure 9 that since 2001, the number of set pair potential publications in CNKI and WOS generally showed a fluctuating upward trend, with rapid growth after 2018. In data, the number of papers published in CNKI is significantly higher than that in WOS. In 2008, the papers on set pair potential began to appear in WOS, and the annual continuous papers appeared only after 2014. The total number of publications showed that the trend of water resources set pair potential publications is the same as that of the total publications. After the subtraction, set pair potential was proposed in the study of water resource carrying capacity in 2018, the proportion of water resources set pair potential research papers in the total set pair potential publications has increased significantly. In particular, the proportion of water resources set pair potential research papers in 2020–2022 has exceeded 78% for three consecutive years, and the highest proportion in 2021 is 91%. The set pair potential has gradually expanded to more research fields.

As shown in Figure 10a, in CNKI, set pair potential has been divided into two categories: traditional set pair potential, and subtraction set pair potential. Subtraction set pair potential has developed into an important development direction. Figure 10b shows that the set pair potential of water resources is mainly concentrated in the evaluation of water resource carrying capacity, trend analysis, and drought disaster risk assessment. The subtraction set pair potential is mainly applied, and the semi-subtraction set pair potential and other connection number adjoint functions are developed. In WOS, there are still very few papers on the set pair potential of water resources. The research mainly focuses on the evaluation and diagnosis of water resource carrying capacity and the comprehensive evaluation of the water resources complex system. In general, the current research on set pair potential analysis of water resources is mainly applied to the related fields of water resource safety evaluation. In the future, it can be widely applied to engineering safety, transportation, risk assessment, ecological environment assessment, program decision-making, and other research fields.

#### 4.2.1. Division Set Pair Potential

The general set pair potential calculation Equation (4) proposed by Mr. Zhao Keqin, also known as the division set-pair potential, is limited in practical applications such as hydrological water resources system engineering because the calculation of the uncertainty term *b* is not directly embodied in the equation and is constrained by the constraint that *c* ≠ 0. When *c* tends to 0, it may lead to a very small difference in the connection number with a large difference in the set pair potential, or even the set pair potential tends to be positively infinite.

Based on the division set-pair potential, to further overcome the lack of consideration of discrepancy information in Equation (4), the pessimistic set pair potential is constructed by assuming that the set migrates to the most unfavorable state within the system, and transferring all the uncertainty terms to the opposing terms [49]:

(13) $$shi{\left(H\right)}_{P}=a/(b+c)$$

where b + c ≠ 0. Similarly, the optimistic set pair potential can again be constructed by considering the most favorable state migration within the set pair system and transferring the uncertainty terms all to the same term [50]:

(14) $$shi{\left(H\right)}_{O}=(a+b)/c$$

where c ≠ 0. It showed that the pessimistic set pair potential and the optimistic set pair potential are two extreme values of the division set pair potential, which together constitute the interval of the division set pair potential [*shi*(*H*)_P_, *shi*(*H*)_O_]. Although they both take into account the influence of uncertain information, it is still impossible to avoid the lack of mathematical significance of set pair potential change and the constraints of practical application when the denominator is close to 0.

#### 4.2.2. Exponential Set Pair Potential

To solve the constraint that the denominator is zero and the set pair potential loses its mathematical meaning in the calculation of the division set pair potential, Li et al. proposed the concept of generalized set pair potential using an exponential function instead of division (also known as exponential set pair potential) [51]:

(15) $$shi{\left(H\right)}_{e}=e^{a-c}$$

Further considering the influence of the difference degree term on the set pair potential, Li et al. [51] further proposed the generalized set pair pessimistic potential and the generalized set pair optimistic potential:

(16) $$shi{\left(H\right)}_{ep}=e^{(a+b)-c}$$

(17) $$shi{\left(H\right)}_{eo}=e^{a-(b+c)}$$

It suggested that the exponential set pair potential overcomes the deficiencies of the division set pair potential with the denominator of 0, but at the same time, it also changes the dimensional properties and quantitative change relationship of the original connection components *a*, *b*, and *c*. The interval range becomes [1/e, e], which is also different from the value interval of the original connection number on [−1, 1]. It is different from the connotation of the relationship degree judgment of the original connection number to a certain connection, which limits its application in the analysis of water resources system problems. Currently, it has been applied to the analysis of the vulnerability of water resource systems [52] and classification of runoff [53], while other problems of water resource systems are less applied.

#### 4.2.3. Subtractive Set Pair Potential

To further overcome the obstacles that the denominator of the division set pair potential is 0, the value range of the exponential set pair potential and the dimensional properties are inconsistent with the original meaning of the connection number. Jin et al. [22] fully considered the influence of the original meaning of the set pair potential and the uncertainty of the connection number, and proposed the subtraction set pair potential of each connection number component in 2018:

(18) $$shi{\left(H\right)}_{s}=a+ba-c-bc=(a-c)(1+b)$$

The range of the subtraction set pair potential is [−1, 1], which is consistent with the range of the connection number, and maintains the dimensional properties and order of magnitude of the connection components *a*, *b*, and *c* in the original connection number. According to the “principle of equalization”, the subtraction set pair potential can be divided into five levels [22]: anti-potential [−1, −0.6), partial anti-potential [−0.6, −0.2), balance potential [−0.2, 0.2], partial same potential (0.2, 0.6], and same potential (0.6, 1.0]. The subtraction set pair potential overcomes the main shortcomings of the division set pair potential and the exponential set pair potential, and directly reflect the overall macro relative deterministic development trend of the connection number. It has been rapidly applied in the evaluation and diagnosis of water resource carrying capacity [54,55], spatial equilibrium evaluation of water resource carrying capacity [56], evaluation and diagnosis of water resources exploitation intensity [57], and other fields.

Correspondingly, the subtractive set pair potentials [58] (Equation (19)) for four-element connection number and (Equation (20)) five-element connection number [48] were proposed:

(19) $$shi{\left(H\right)}_{s4}=(a-c)(1+b_{1}+b_{2})+0.33(b_{1}-b_{2})(b_{1}+b_{2})$$

(20) $$shi{\left(H\right)}_{s5}=(a-c)(1+b_{1}+b_{2}+b_{3})+0.5(b_{1}-b_{3})(b_{1}+b_{2}+b_{3})$$

where 0.33 in Equation (19) and 0.5 in Equation (20) are both conversion coefficients for transforming the values of the partial same degree of difference (partial degree of opposite) terms into the values of the sameness degree (the opposites degree) terms. Similarly, it can also be derived to the subtraction set pair potential of other multivariate connection numbers.

Also, some scholars have carried out comparative studies between subtractive set pair potential and other methods. Li et al. [59] comprehensively compared and evaluated the five-element subtractive set pair potential, logistic, and TOPSIS methods. It was found that the logistic model only considers the degree of attribution, which is greatly influenced by the weights, and that the TOPSIS model is not suitable for a single index, and both models have some limitations. The five-element subtractive set pair potential not only evaluates the weights of different indicator levels, but also the fuzzy relationship between the indicators and the evaluation level criteria. The value of the subtractive set potential can also be used as a diagnosis of obstacle indicators. It is the most suitable comprehensive evaluation method among the three models.

**Figure 11:**
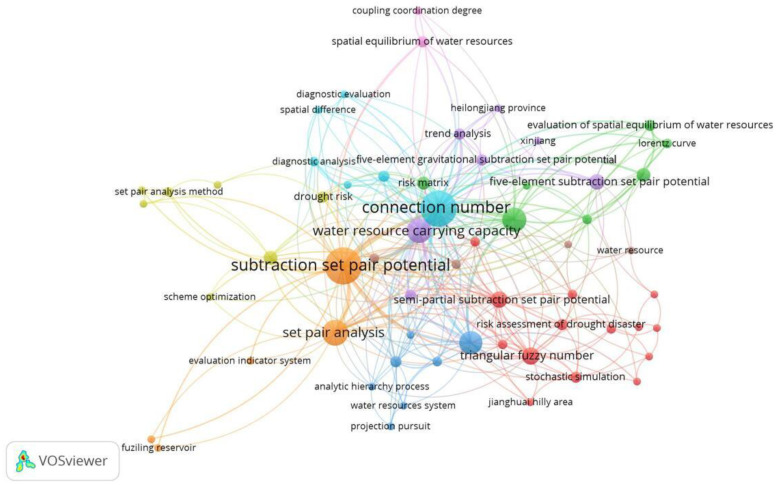
Keywords co-occurrence labelling map of subtraction set pair potential in CNKI database.

In CNKI, we conducted an advanced search with the theme of “subtractive set pair potential”, and applied the VOSviewer 1.6.19 tool to analyze the keywords of the research hotspots of subtraction set pair potential (Figure 11). It was found that since the proposal of subtractive set pair potential in 2018, there have been nearly 100 relevant papers published in less than five years, mainly focusing on the research on the evaluation of the carrying capacity of water resources [22,60], combining with the Gini coefficient, the risk matrix, and other methods to expand the relevant research to the spatial equilibrium of water resources [48,61], the assessment of the risk of drought [56] and other areas of research.

The three-element subtraction set pair potential is quickly extended to four-element [47] (Equation (19)), and five-element [48] (Equation (20)) subtraction set pair potential. The application is also extended to other areas such as underground reservoir risk assessment [62], water conflict risk assessment [63], river health evaluation [64], and environmental capacity study [65]. Some scholars compared the three methods of logistics, TOPSIS, and subtraction set pair potential and found that the later has higher accuracy [65].

Meanwhile, the gravitational subtractive set pair potential is constructed by combining with the principle of gravity in physics and applied to the fields of drought risk assessment [66], water resource carrying capacity evaluation [67], water resources vulnerability evaluation [68], etc. Coupled with the semi-partial connection number, the semi-partial subtraction set pair potential [37] is successively constructed, while continuous semi-partial subtraction set pair potential [69], double semi-partial subtraction set pair potential [70], and continuous evolutionary semi-partial subtraction set pair potential [71] are developed, respectively. The coupled semi-biased subtractive set-potential functions are more reasonable than the traditional subtractive set-potential in characterizing the transformational relationship of the connection components. The continuous semi-partial subtraction set pair potential and the double semi-partial subtraction set pair potential use the transformation relationship between adjacent connection components to solve the problem that the coefficients of the uncertainty term *b* depend on the empirical value in the semi-partial subtraction set pair potential method from different points of view. The continuous evolution semi-subtraction set pair potential further fully exploits the existing values of the connection components and the information on the relationships between the adjacent and non-adjacent connection components, which fully reflects the mutual transformation relationship of the uncertain terms.

It showed that the cross-application of a variety of adjoint functions to reveal the dialectical evolution characteristics of the same–difference–opposite relationship can further improve the applicability of the connection number method. In addition to the application of water resources research, it can also be extended to the evaluation of complex systems such as resources and environment, ecology, trend analysis, and disaster risk assessment.

### 4.3. Partial Connection Number

**Figure 12:**
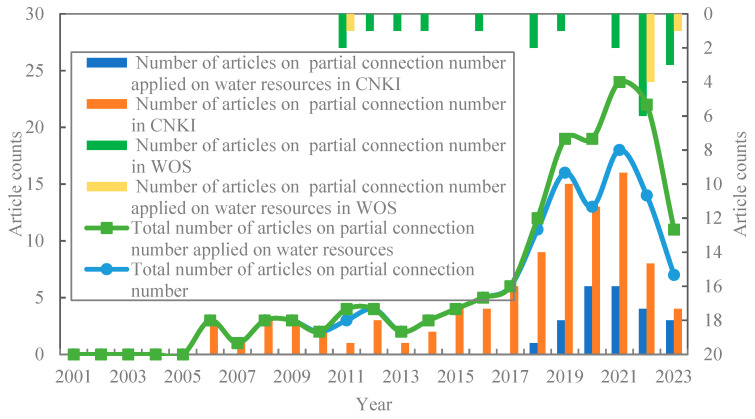
Statistics of the number of partial connections in CNKI and WOS.

In CNKI, an advanced search was carried out themed “partial connection number”. There were 98 relevant papers coming out until October 2023, from which 23 papers related to water resources were screened out. In WOS, a search was carried out with “partial connection number” as the theme, a total of 20 papers were searched, and 6 papers related to “water resources” were screened and analyzed statistically (Figure 12). The VOSviewer 1.6.19 tool was applied to generate keyword network visualization and analysis diagrams of partial connection numbers (Figure 11).

As seen from Figure 12, after the partial connection number proposed in 2005, the number of publications generally showed a fluctuating upward trend. Before 2017, the number of publications was relatively small, and then showed a rapid growth. In 2021, the number of publications was the highest, reaching 18, and decreased in 2022; the total number of papers published in CNKI is significantly higher than that in WOS, and there is a great deal of space for the application research of partial connection number to expand abroad.

**Figure 13:**
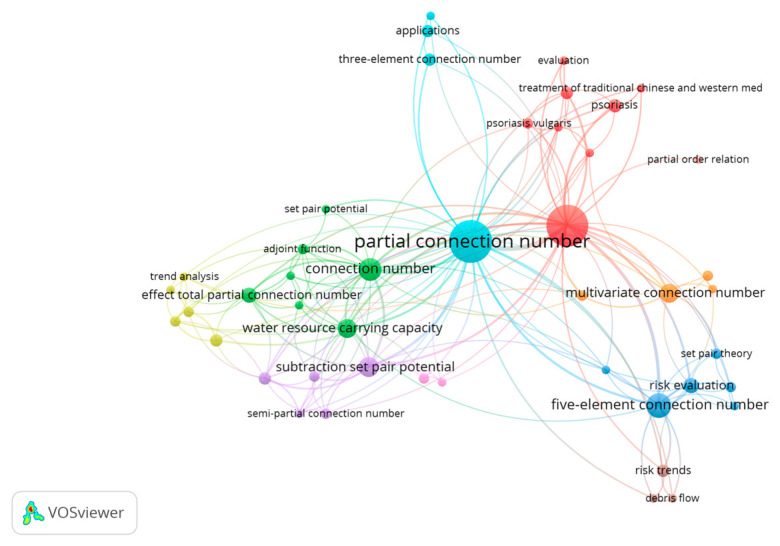
Keywords co-occurrence labelling map of partial connection number in CNKI database.

**Figure 14:**
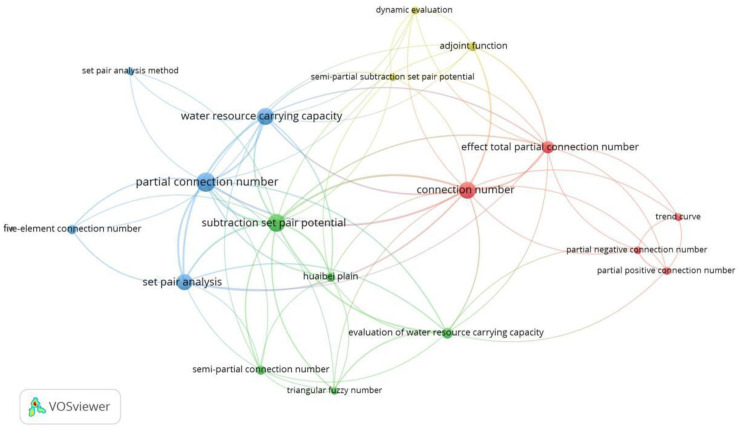
Keywords co-occurrence labelling map of partial connection number for water resources in CNKI database.

As shown in Figure 13 and Figure 14, there are three main application fields of the current partial connection number: water resources, medical diagnosis (psoriasis), and trend and risk analysis. The application of partial connection number in the field of water resources research is mainly for water resource carrying capacity evaluation research, and the adjoint functions such as total partial connection number and semi partial connection number are developed, coupled with subtraction set pair potential for the evaluation and diagnosis of water resource carrying capacity.

#### 4.3.1. Total Partial Connection Number

The total partial connection number includes the partial positive connection number and partial negative connection number, which portray the contradictory movement of the positive and negative migration of each connection component at the micro level, and characterize the direction and intensity of the contradictory movement [31]. It can also reflect the change of the uncertainty state in the set pair event and judge the development trend of the event.

At present, the calculation of total partial connection number has not been unified, and the more used expressions for partial connection number are mainly of the following three types.

Qin et al. [72] considered the total partial connection number expression for the three-element connection number as:

(21) $$\partial \mu_{1}=\partial^{+}\mu_{1}-\partial^{-}\mu_{1}=\frac{a}{a+b}+\frac{b}{b+c}{i_{1}}^{+}-(\frac{b}{a+b}+\frac{c}{b+c}{i_{1}}^{-})$$

where ${i_{1}}^{+}=\partial a/(\partial a+\partial b)$.

Lu et al. [73] considered the total partial connection number expression for the three-element connection number as:

(22) $$\partial \mu_{2}=\partial^{+}\mu_{2}+\partial^{-}\mu_{2}=\frac{a}{a+b}+\frac{b}{b+c}{i_{2}}^{+}+\left(\frac{b}{a+b}{i_{2}}^{-}+\frac{c}{b+c}j\right)$$

where ${i_{2}}^{+}=\partial a/(\partial a+\partial b)$, ${i_{2}}^{-}=\partial^{-}c/(\partial^{-}b+\partial^{-}c)$, *j* = −1.

It suggested that from the two equations above, compared with Equations (7) and (21), they divide the coefficient of difference *i* into *i*^+^ and *i*^−^, and the coefficient *i*^−^ is moved from *b*/(*a* + *b*) position to the position of the *c*/(*a* + *b*), excluding the coefficient *j*, instead of the difference between the partial positive connection number and the partial negative connection number to represent the total partial connection number, which has an obvious deviation from the original meaning of Equation (7). Equation (22) also divides the difference coefficient *i* into *i*^+^ and *i*^−^ to distinguish the difference between positive and negative connection numbers. At the same time, the influence of the index coefficient *j* is considered, but the influence of the positive and negative values of the difference coefficient *i*^−^ in the partial negative connection number is not considered.

Therefore, Jin et al. [24] fully combined the connotation of the partial positive connection number, compared them to give the meaning of the partial negative connection number, and proposed the effect total partial connection number:

(23) $$\partial \mu_{3}=\partial^{+}\mu_{3}+\partial^{-}\mu_{3}=\frac{a}{a+b}+\frac{b}{b+c}{i_{3}}^{+}+\left(\frac{b}{a+b}{i_{3}}^{-}+\frac{c}{b+c}j\right)$$

where ${i_{3}}^{+}=\partial a/(\partial a+\partial b)$, ${i_{3}}^{-}=-\partial^{-}c/(\partial^{-}b+\partial^{-}c)$, *j* = −1.

The obvious difference in Equation (22) is that the negative effect of the partial negative connection number is distinguished by referring to the partial positive connection number and its positive effects, and the *i*_3_ in Equation (23) takes positive and negative values according to the positive and negative effects, respectively. Jin et al. further compared the size of the three (∂*μ*_1_, ∂*μ*_2_, ∂*μ*_3_), and compared Equation (23) with the subtraction set pair potential by random simulation with very small errors. It has been applied to the study of water resource carrying capacity [24] and emergency management capability evaluation of natural disasters [74]. It is believed that the application of the effect total partial connection number is more stable and reasonable.

In addition to the three-element partial connection number, it can also be extended to the partial connection number of four, five, and more element connection numbers, and even from the first order to the second order and higher orders [75,76]. However, currently, the development of higher-order partial connection numbers is very slow. On the one hand, lack of theoretical support for the expansion of partial connection numbers from first-order to higher-order, and also lacking the analysis of its physical connotation; on the other hand, the calculation equation of high-order total partial connection number is still very controversial. The meaning of the calculated value, the verification method, and the practical application are all inadequate, which seriously limits the development of high-order partial connection numbers.

It is suggested that the calculation expression and connotation of the partial connection number have not yet reached a consensus [12]. The meaning of the connection component term before and after the revolution, the meaning of the difference coefficient in the calculation formula of the partial positive and partial negative connection number and its relationship with the second-order partial connection number, and the meaning of the calculated value of the total partial connection number, and other key issues, have not been fully unified. There are few ways to verify the total partial connection number, and the theoretical research on the high-order partial connection number is even more insufficient. The research of the partial connection number needs to be further studied through the basic connotation analysis to the computational thinking path of quantitative calculation.

#### 4.3.2. Semi-Partial Connection Number

The semi-partial connection number can reveal the contradictory movement of the connection number components at the micro level and quantitatively express the mutual migration and transformation (dynamic evolution) of the connection number components in the system structure. The semi-positive connection number of the connection number components *a* and *b* of the three-element connection number *u* = *a* + *bi* + *cj* [38,40]:

(24) $$\partial^{+}a=\frac{a}{a+b},\partial^{+}b=\frac{b}{b+c}$$

It can be considered that the current *a* is originally at the *b* level, and migrates from the *b* level to the positive same migration rate, and the current *b* is originally at the *c* level and migrates from the *c* level to the positive different migration rate. Similarly, there is a semi-negative connection number of the connection number components *a* and *b* [39]:

(25) $$\partial^{-}b=\frac{b}{a+b},\partial^{-}c=\frac{c}{b+c}$$

The semi-partial connection number is used to describe the mutual migration movement between the connection number components and obtain the corrected connection number components, which further enhance the rationality and accuracy of the connection number calculation. It has been used in the evaluation of water resource carrying capacity [41,77], drought risk assessment [72], water resource spatial equilibrium [78], and so on.

### 4.4. Adjoint Function Methods for Other Opposite Types of Connection Number

The positive-negative [1 × (−1)] connection number is the most commonly used type of connection number. In addition, there are inverse (reciprocal) type [*k* × (1/*k*)] connection number, mutually exclusive (with or without) type (*k* × 0) connection number, complementary type (*k*_1_ + *k*_2_ = 1) connection number, and virtual-real type (*k* ↔ *k*′) connection number [79]. Among them, the positive–negative connection number can be regarded as a union of the inverse connection number and its connection number in the negative direction. The complementary connection number is regarded as a proper subset of the inverse connection number. The inverse type, positive–negative type, mutually exclusive type, and complementary type connection number are all connection numbers that reflect the objective existence of opposite relations, and can be regarded as a true subset of the virtual–real connection number. The positive–negative connection number and its adjoint functions have a wide range of applications and are the mainstream of current applications, while other types of connection numbers are in the conceptual stage. The calculation equation of connection number is yet to be explored, and the adjoint function is rarely reported. By referring to the existing adjoint functions of positive–negative connection numbers, the adjoint functions of other types of opposite connection numbers can be developed, which can further describe, mine, and utilize various uncertain information in the complex system of water resources to achieve information empowerment.

## 5. Conclusions and Prospects

Set pair analysis is intuitive, profound, and simple to calculate from the dialectical relationship to deal with the uncertainty characteristics. After more than 30 years of development, it has made a lot of important progress in the field of theoretical research and applied research. Especially in the past 20 years, the adjoint function of connection number has become an important frontier and research hotspot of set pair analysis, and it is particularly active in the field of complex water resources systems, some new adjoint functions such as subtraction set pair potential, semi-partial connection number, and effect total partial connection number are proposed in the field of water resources applications. Based on the research and analysis of the theoretical connotation and application practice of the set pair analysis connection number and its adjoint function, we focused on the theory and methodology of the development of the adjoint function of the set pair analysis connection number of water resources, analyzing and summarizing the development and application of the adjoint function of the connection number in the research of water resources in the past 20 years using the bibliometric method, further promoting the development of the adjoint function of the set pair analysis. Although descent achievements have been made in the investigation of the adjoint function of set pair analysis, there are still some problems, such as relatively weak theoretical basis, insufficient practical experience, insufficient application field, and insufficient international promotion, waiting for further exploration and evaluation.

### 5.1. Conclusions

(1) The development of water resource set-pair analyses is rapid, but the theoretical basis is still relatively weak, and the promotion of the set pair analysis method to international research is insufficient. Since 2001, the number of papers on set pair analysis of water resources has shown a fluctuating growth trend, and the number of domestic papers is significantly higher than that of foreign papers. The development process from 2001 to 2022 can be divided into three stages: before 2005, the fundamental theory of set-pair analysis began to be introduced into the analysis of water resources systems, and the development of water resource set pair analysis research was relatively slow; from 2005 to 2017, the emergency of partial connection number boosted the expansion of water resource set pair analysis research; after 2018, the adjoint functions such as subtraction set pair potential and semi-partial connection number further elevated a new round of rapid development in the research field of water resource carrying capacity and drought disaster risk assessment. From the perspective of research disciplines, the research on set pair analysis in water resource complex systems mainly focused on the field of water conservancy and hydropower engineering and resource science, followed by environmental science and resource utilization. The comprehensive evaluation is widely used in the research and application of water resource set pair analysis, but the research in the fields of prediction and decision-making is relatively insufficient. The SPA research is mainly concentrated in China, with great potential to be promoted abroad.
(2) The development of the adjoint function in set pair analysis of water resources is unbalanced. Set pair potential and partial connection number are the most important research directions of the adjoint function of connection number. The subtraction set pair potential has developed rapidly into an important area of research, while the research on connection entropy are relatively deficient, which needs to be further strengthened, and the situation on neighboring connection numbers is even worse. The coupling of set pair potential and partial connection number to construct new adjoint functions develops rapidly, as it reveals the dialectical evolution characteristics of the same, different, and opposite relationship of connection number more deeply. The research branch of coupled adjoint functions such as semi-subtractive set pair potential has been developed, which may become an important development course in the future. The adjoint function of connection number is widely used in the field of water resources, while most of the research focuses on the evaluation of water resource carrying capacity, risk assessment, and water safety evaluation.
(3) The coupling adjoint function of the connection number develops rapidly and necessitates further exploration. The adjoint functions, such as set pair potential, partial connection number, and coupled adjoint function are the primary field of research of connection number in water resource set pair analysis. Among them, the set pair potential adjoint function can be divided into three categories: division set pair potential, exponential set pair potential, and subtraction set pair potential, where the expression forms, dimensional properties, and order of magnitude of the three categories are different. The subtraction set pair potential remains the original dimension and quantity variation range of the connection number and is broadly used, which can be combined with the universal gravitation to construct the gravitational subtraction set pair potential. Coupled with the semi-partial connection number, a series of new connection number adjoint functions, such as semi-partial subtraction set pair potential, continuous semi-partial subtraction set pair potential, double semi-partial subtraction set potential, and continuous evolutionary semi-partial subtraction set pair potential, have been developed successively, which have rapidly grown into a relatively independent research direction. The transformation of adjoint functions, such as semi-partial connection numbers at the micro level of the connection component, can be fully explored to construct a new adjoint function.
(4) The evolution of total partial connection numbers and high-order partial connection numbers are limited by the lack of clarity of physical connotation, while that of semi-partial associations is more rapid. There are two main categories of partial connection number: total partial connection number, and semi-partial connection number. To date, there is still no consensus on the first-order calculation expression and connotation of the total partial connection number, the main reason for which is that the positive and negative values of the difference coefficient in the calculation equation of partial positive and partial negative connection number, the value of the opposite degree coefficient, and the addition and subtraction of partial positive and partial negative connection number are controversial. The effect total partial connection number is a relatively reasonable calculation method at present. The analysis of the physical connotation of the higher-order partial connection number is insufficient, the theoretical research is lacking, and the development is relatively slow. The semi-partial connection number can describe the mutual migration movement between the connection number components, further modify the connection number components, enhance the rationality and accuracy of the connection number calculation, and be combined with the set pair potential for rapid application in the fields of water resource carrying capacity evaluation and drought risk assessment.
(5) The connection number and its adjoint function of different types of opposite relations need to be expanded. The positive–negative connection number develops rapidly due to its wide range of applications, while the inverse (reciprocal) connection number, mutually exclusive (with or without) connection number, complementary connection number, virtual-real connection number, and other types of connection number develop slowly; also, the corresponding adjoint function is rarely reported. Concerning the adjoint functions of positive and negative connection numbers, the adjoint functions of the above four types of connection numbers can be constructed to comprehensively explore various uncertain information in the complex system of water resources.

### 5.2. Prospects

(1) In light of the issue regarding the weak theoretical foundation of set pair analysis, it is imperative to provide a more precise explanation and conceptual definition of the adjoint function in relation to its original significance. The adjoint function of the connection number is a structural function based on the variables such as the connection components *a*, *b*, *c*, and the difference coefficient *opposite* and opposite coefficient *j*. It is a state function that reflects the interaction between the connection components, and a characteristic function that depicts the relative development trend of the set pair to the event. Therefore, when constructing the adjoint function, two aspects should be fully considered: first, the concept boundary of the adjoint function is clear, and the structure of the same, different and opposite is complete, reflecting the interaction relationship between the connected components and the overall trend of the set of events in general; second, the adjoint function maintains the consistency of its dimensional properties and order of magnitude variation characteristics with the connection number. For example, the construction of the set pair potential adjoint function can not only consider the same degree *a* and the opposite degree *c*, but also needs to include the difference degree *b* to construct the associated adjoint function, which comprehensively reflects the determination and uncertainty characteristics of the set pair. At the same time, the constructed adjoint function is used as the characteristic function of the connection number, and its dimension and quantity change range should be consistent with the connection number, which is more convenient for analyzing the characteristics of the set pair.
(2) The research on the adjoint function of water resources connection number is largely focused on the field of comprehensive evaluation, and the research on water resources set pair prediction and decision-making is relatively lagging. At present, the set pair prediction of water resources is mainly single-factor prediction and statistical prediction, and it is necessary to strengthen the research on the set pair prediction model that integrates the physical mechanism model of water resources. Meanwhile, it is critical to gradually shift from single-factor prediction to complex water resources system prediction and early warning research in the field of water resource carrying capacity, water resource security, and so on. The research on set pair decision-making of water resources belongs to the category of comprehensive evaluation. In multi-index decision-making, it may lead to different difference coefficients corresponding to different decision-making schemes. Therefore, it is necessary to carry out quantitative decision-making model research based on the combination of set pair analysis and simulation of water resource complex systems. Also, the dynamic decision-making of water resources can be further studied by combining the set pair potential, partial connection number, and other adjoint functions.
(3) Given the inconsistency of the calculation method of the adjoint function, it is crucial to combine the practical application of the adjoint function of the connection number to further improve the construction of a universal and relatively unified calculation method of the adjoint function. Currently, there are many different forms of expression of the same adjoint function (such as set pair potential, total partial connection number, etc.), however, subjective assumption occurs in its construction, due to the lack of unified rules, leading to different results in the practical application of different fields. Currently, a unified conclusion is absent in the physical connotation interpretation of different forms of adjoint function. For example, there are different types of adjoint functions of set pair potentials, such as division, subtraction, and exponential set pair potential, and different formulas for calculating the total partial connection numbers Therefore, introducing a unified and perfect expression for the calculation of the adjoint function and the clarification of its application conditions becomes an urgent issue to be solved at present.
(4) At present, the development of the adjoint function of higher-order partial connection number, adjacent connection number, and connection entropy is relatively slow, while the calculation formula is controversial. Therefore, it is essential to perform in-depth research on the value of difference coefficients and opposition coefficients in partial connection numbers, the operation research of partial positive connection numbers and partial negative connection numbers, and carry out the expansion research of first-order to higher-order partial connection numbers in due course. Also, research on the construction of a unified adjacent connection number and connection entropy concomitant function with clear physical meaning should be conducted.
(5) The adjoint functions such as the partial connection number, the semi-partial connection number, and the subtraction set pair potential can fully reflect the mutual transformation relationship between the connection components from the micro-scale. The cross-coupling of different adjoint functions can further reveal the dialectical evolution characteristics of the same–difference–opposite relationship, continuously strengthen the research of set pair analysis theory and method, and improve the applicability of the connection number method in the analysis of complex water resources system problems. Meanwhile, considering the adjoint function of the positive and negative opposite connection numbers, the adjoint functions of other different types of opposite connection numbers can be constructed, and various uncertain information in the complex system of water resources can be fully excavated and utilized.
(6) The development of set pair analysis in the direction of water resources is not balanced. The research and application of the adjoint function are primarily conducted in China, while the promotion to other countries faces great potential and challenges. It is suggested that firstly organize international conference on set pair analysis research or sub-conference on water resource set pair analysis topics and promote the latest research results of set pair analysis. Secondly, carry out set pair analysis course training to assist more people in quickly mastering the methods through thematic learning. Thirdly, encourage cooperative research in different fields to promote set pair analysis and adjoint functions to carry out the research and application of evaluation, diagnosis, prediction, and decision-making in other fields such as engineering safety, transportation, ecological environment, disaster risk, and scheme selection. Set pair analysis is expected to attract more extensive international attention and application in the future.

## Data Availability

The data presented in this study are available in the Web of Science and CNKI.
